# The Outcome of Octogenarian Patients with Multiple Myeloma Treated Outside Clinical Studies, Focusing on Tolerability and Efficacy of Treatment

**DOI:** 10.3390/cancers16193329

**Published:** 2024-09-29

**Authors:** Dana Amsterdam, Ori Grossberger, Natan Melamed, Dor Shpizer, Svetlana Trestman, Tamir Shragai, Yael C. Cohen, Irit Avivi

**Affiliations:** 1 Hematology Division, Tel Aviv Sourasky Medical Center, Tel Aviv 6423906, Israel; orig@tlvmc.gov.il (O.G.); natanm@tlvmc.gov.il (N.M.); shpitzerd@gmail.com (D.S.); svetlanat@tlvmc.gov.il (S.T.); tamirsh@tlvmc.gov.il (T.S.); yaelcoh@tlvmc.gov.il (Y.C.C.); 2 Faculty of Medicine, Tel Aviv University, Tel Aviv 6139001, Israel

**Keywords:** multiple myeloma (MM), octogenarian patients, individualized therapy, treatment tolerability, adverse events (AEs), sequential therapy

## Abstract

**Simple Summary:**

There is a significant gap in research and guidelines for treating octogenarian multiple myeloma (MM) patients. This retrospective study examined 101 MM patients, median age 84 years (80–98), treated outside clinical studies at TASMC between 2010 and 2023, aiming to review real-world practices and outcomes experienced by this vulnerable group of patients. Of these patients, 87% received a bortezomib-based regimen; 20% received lenalidomide ± bortezomib; 44% were treated with novel agent-based doublets, and 51% with triplets/quadruplets. Despite the employment of reduced doses of steroids and lenalidomide, treatment-related toxicity was high, including 9% who suffered grade 5 events. Of these patients, 67% received subsequent lines, resulting in an impressive median overall survival of 42 months (1–141) in the entire cohort. These results highlight the effectiveness of personalized therapy for octogenarian multiple myeloma (MM) patients and emphasize the need for further real-time studies to establish guidelines for the management of this vulnerable and growing population.

**Abstract:**

**Background:** Data on the outcome of octogenarian multiple myeloma (MM) patients (pts), especially if treated outside clinical studies, are scanty. **Aims and Methods**: MM pts ≥ 80 years, treated at TASMC with first-line therapy between 2010 and 2023, were reviewed. Characteristics and outcomes were analyzed. **Results:** A total number of 101 pts, of whom 54 were males with a median age of 84 years (80–98), were included. Among them, 67.4% had a Charlson comorbidity index of ≥5, 37% had ISS-3 (International staging system) and 20% had Revised-ISS-3. In our study, 44.5% received doublets and 50.5% received triplets/quadruplets. A bortezomib-based regimen was applied in 87%, and IMiDs were used in 27.7%. Despite an upfront employment of a low lenalidomide dose, dose reductions were required in 48%. Grade ≥ 3 adverse events (AEs) (mainly infections) were documented in 36.6% of patients, including grade 5 events in 9%, all attributed to infections. The overall response rate was 69%, including 31% ≥ VGPRs (Very good partial response). Sixty-seven percent (67%) received second-line therapy, administered within a median period of 12 months (1–84). Within a median follow-up period of 36 m (1–141), the median overall survival (OS) approached 42 m (range: 1–141); being shorter in pts > 84 years (HR = 1.7, *p* = 0.03), pts with lung disease (HR = 1.8, *p* = 0.044) and pts with ISS = 3 and R-ISS = 3 (HR = 1.65, *p* = 0.0016 and HR = 2.45, *p* = 0.006, respectively); **Conclusions**: Octogenarians treated outside clinical studies often have a lower tolerance to treatment. Nevertheless, upfront administration of low doses of anti-MM agents provided a response in the majority of patients, translated into impressive OS. Nevertheless, mortality due to AEs was high, emphasizing the need for new, “octogenarian-oriented” treatment protocols.

## 1. Introduction

Multiple myeloma (MM) predominantly affects older adults. The incidence increases substantially with advancing age, particularly among individuals aged 80 years and older, commonly referred to as octogenarians. The management of MM in octogenarian patients presents unique challenges due to several factors, including age-related physiological changes, the high incidence of concomitant comorbidities, and reduced tolerance to treatment [1,2]. Despite advancements in MM therapy, data on octogenarian MM patients remain limited, primarily due to their underrepresentation in clinical trials. Furthermore, the majority of available data on treatment of MM stem from prospective trials wherein participants typically exhibit a higher level of fitness than their real-world counterparts, who may fail to meet the stringent inclusion criteria of such studies. This discrepancy results in a notable gap, especially in our comprehension of MM within the elderly demographic [3,4,5,6].

The exclusion of frailer or medically complex individuals from prospective trials limits the generalizability of their results and hampers our ability to formulate evidence-based recommendations for managing MM in the elderly population.

This underscores the urgent need for research endeavors that encompass a broader spectrum of octogenarian MM patients, including those with comorbidities and functional limitations, to provide a more comprehensive understanding of the disease course and management and optimize clinical care strategies tailored to the needs of this vulnerable population.

The current study aimed to define the clinical characteristics and treatment patterns, including tolerability to treatment and treatment modifications, and reviewed the outcomes of octogenarian MM patients, with the goal of informing personalized treatment strategies and improving patient care in this age group.

## 2. Materials and Methods

This study aimed to investigate the characteristics and outcomes of octogenarian MM patients who initiated first-line therapy at Tel Aviv Sourasky Medical Center (TASMC). Data were reviewed for all consecutive MM patients aged ≥ 80 years, diagnosed and considered as requiring an anti-MM treatment, between 2010 and 2023. Approval was obtained from the local institutional review board at Tel aviv Sourasky medical center (IRB number #0264-24). Inclusion criteria comprised age ≥ 80 years, a confirmed histological diagnosis of MM, the existence of CRAB- SLIM criteria [7], and no prior lines of anti-MM therapy. Patients diagnosed with smoldering MM were excluded.

Data regarding demographics, clinical characteristics, MM-related risk factors, treatment regimens being given, and doses of each anti-MM agent were recorded. Treatment-duration causes for discontinuation of treatment and treatment-related toxicities were reviewed. The following parameters were assessed: response to therapy determined according to the International Myeloma Working Group (IMWG) criteria [8], administration of subsequent lines of anti-MM therapies, time to next treatment (TTNT) (assessed as the time from initiation of first-line treatment to the initiation of second line treatment), overall survival (OS), and causes of death.

### Statistical Analysis

Categorical variables were described as frequency and percentage. Continuous variables were evaluated for normal distribution using histograms. Normally distributed continuous variables were described as mean and standard deviation, while non-normally distributed variables were described as median and interquartile range. The chi-square test and Fisher’s exact tests were used to study the association between categorical variables and overall response rate (ORR), and independent-sample T-tests and Mann–Whitney tests were applied to study the association between ORR and continuous variables. OS and TTNT were measured from diagnosis. Kaplan–Meier curves were used to describe events during the follow-up period and to evaluate the median survival time. The reverse censoring method was used to report the median follow-up time. Univariate and multivariate Cox regression analysis was used to evaluate the association between each predictor and the study outcomes of TTNT and OS. Hazard ratios (HRs) with 95% confidence intervals (CIs) were reported. All statistical tests were 2-sided. *p* values smaller than 0.05 were considered statistically significant. R was used for our statistical analyses (R version 4.3.2 (2023-10-31 ucrt) Tel Aviv, Israel).

## 3. Results

### 3.1. Patient Characteristics

A total number of 101 patients, 54 males, were included in the study. The median age at MM diagnosis was 84 years (80–98). Almost all patients had concomitant comorbidities, including 27% (n = 27) which had chronic renal failure (CRF) attributed to prior comorbidities such as hypertension and diabetes (n = 23), diabetes alone (n = 1) and idiopathic CRF (n = 3). Sixty-seven (67%) percent (n = 68) presented with a Charlson comorbidity index equal to or greater than 5. In total, 57% (n = 58) had lytic bone lesions, 19% (n = 19) had MM-related acute renal failure and 49.5% (n = 50) had anemia. International Staging System (ISS)-3 and high-risk Fluorescence in situ hybridization (FISH) were recorded in 55% and in 30% of patients for whom blood and FISH tests were available (38/69 and 23/77, respectively). Table 1 presents the patients’ characteristics. Patients received various agents reflecting physical choice and the reimbursement policy in Israel at the time of initiating treatment (Bortezomib approved for first line since 2014, lenalidomide since 2019, and daratumumab since 2023). In total, 88 patients received a bortezomib-containing regimen, while 28 patients received an IMID-containing regimen (27 with lenalidomide and 1 with thalidomide). Within these two groups, 20 patients were treated with a combined PI-IMID regimen. Doublets were used in 44.5% (n = 45), triplets in 48.5% (49), and quadruplets in 2 (2%). Tables S1 and S2 present a detailed review of the different regimens used as first-line and second-line treatments, respectively.

### 3.2. Outcomes and Tolerance to Treatment

#### 3.2.1. Adverse Events and Dose Modifications

Grade ≥ 3 adverse events (AEs) were documented in 36.6% (n = 37), including treatment-related deaths in 9% (n = 9). Most frequent documented AEs ≥ grade 3 included infections (22%), neutropenia (18%—resulting in grade ≥ 3 neutropenic fever in 5%), thrombocytopenia (10%), cardiotoxicity (3%), hypotension (1%), neuropathy (1%), rash (1%), and diarrhea (1%). Grade ≥ 3 infections were reported in 21% (n = 22), including lower respiratory tract infections (LRTIs) in 10% (n = 11), bacteremia in 4% (n = 4), and Corona virus disease 2019 (COVID-19) in 2% (n = 2(. Treatment-related mortality (TRM) was attributed to infections (n = 9), with a majority being lung infection (n = 7).

Despite the initial use of reduced bortezomib doses (equal to or lower than 1.3 mg/m^2^ in 73% of bortezomib-treated patients (n = 65)), 27% (n = 24) still required a dose reduction during their treatment. Similarly, 48% (13/27) of those treated with lenalidomide experienced dose reductions, including 37% (n = 10) among triplet-treated patients and 11% (n = 3) among doublet-treated patients. These reductions occurred despite the use of a reduced starting dose of 10–15 mg/d for 21 days in 60% of lenalidomide-treated patients.

The overall treatment discontinuation rate due to adverse events was 32.5% (33/101), being lower in patients treated with doublets (26% (11/42)) compared with patients treated with triplets/quadruplets (43% (19/44)), though it was not statistically significant.

Univariate analysis, assessing the impact of demographics, disease-related factors, and treatment regimens (doublets vs. triplets/quadruplets) on the risk of developing grade ≥ 3 infections, failed to identify any statistically significant factors including triplet vs. doublets, with the exception of having a high-risk ISS (*p* = 0.00413).

#### 3.2.2. Outcomes

The median follow-up period after first-line therapy was 36 months (1–141). At the last follow-up date, only five patients were still receiving first-line therapy. In total, 53% (n = 54) stopped 1 L therapy due to progressive disease (PD), 32% (n = 33) due to AEs, and 6 due to a new, unrelated medical event. Eighteen percent (n = 18) of patients died during 1 L therapy, eight due to disease progression, nine due to AEs, and one due to an unrelated cause.

The maximal response rate approached 70% (n = 71), including 4% complete remission (CRs) (n = 5), 27% very good partial remission (VGPRs) (n = 28) and 38% partial remission (PRs) (n = 38). Sixty-seven percent (n = 68) received second line therapy, administered within a median period of 12 months since the initiation of first-line therapy (IQR range: 1–84 m). The employment of second-line therapy tended to be greater in those that discontinued first-line therapy due to PD (45/54, 83%), compared with those that discontinued therapy due to AEs (17/33, 52%, *p* = 0.09).

Univariate analysis for factors predicting TTNT demonstrated doublets vs. triplets (HR = 2.2, *p* = 0.003) to be the only factor predicting longer TTNT (18 vs. 8 months) as manifested in Table S3 in the supplementary section.

**Figure 1:**
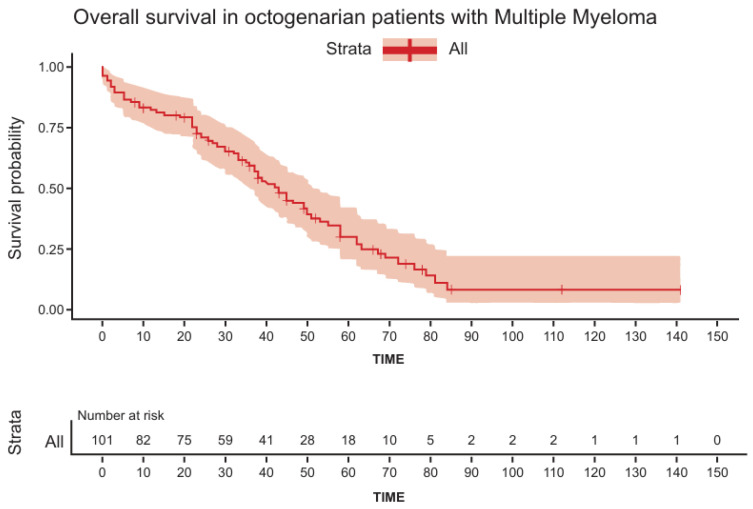
Overall survival in octogenarian patients with multiple myeloma.

**Figure 2:**
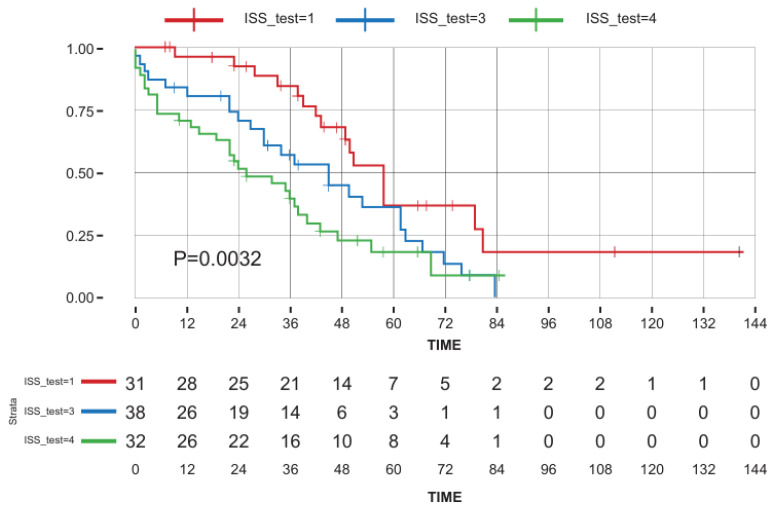
Multivariable analysis for overall survival dependent on ISS. Key: red line—ISS 1–2; green line—ISS 3; blue line—missing data.

The median OS for the entire cohort was 42 m (range: 1–141) (Figure 1), and was found to be shorter in patients > 84 years (HR = 1.7, *p* = 0.03), patients with concomitant lung disease (HR = 1.8, *p* = 0.044), and patients presenting with ISS = 3 and R-ISS = 3 (HR = 1.65, *p* = 0.0016 and HR = 2.45, *p* = 0.006, respectively) (Table 2 presents the univariate analysis and Figure 2 presents OS dependent on ISS). There were no statistical differences in OS between patients treated with doublets vs. triplets (median OS 37 months (1–84 m) vs. median 38 months (1–63 m) (*p* = 0.77)). Multivariate analysis (Table S4) failed to identify any statistically significant factor that predicts OS.

## 4. Discussion

The management of Multiple myeloma(MM) in octogenarian patients presents unique clinical challenges, primarily due to physiological changes associated with aging, a high prevalence of comorbidities, and a general decrease in treatment tolerance. This study provides vital insights into the treatment patterns, tolerability, and outcomes of MM therapy in this demographic, offering a rare glimpse into a group significantly underrepresented in clinical trials. As expected, a significant proportion of our patients had severe comorbidities, evidenced by 67.3% having a Charlson comorbidity index of ≥5. Additionally, 50% had CCT < 30 mL/min, which would typically exclude them from prospective clinical studies. Most patients were treated with bortezomib-based regimens, reflecting the availability of different agents in Israel during the study period and the introduction of first-line lenalidomide only since 2018 [9].

A considerable number of patients received treatment with doublets rather than triplets, balancing efficacy and tolerability—a crucial issue in managing older patients who may have heightened sensitivity to drug-related toxicities. In line with this, the majority of patients started treatment containing a low dose of dexamethasone/prednisone, and those receiving lenalidomide had reduced doses, underscoring the practice of dose adjustment in this age group to mitigate adverse effects. This approach emphasizes that in real-life practice, treatment is often adjusted upfront, indicating that many of these patients might not be presented for clinical studies due to concerns about their ability to tolerate standard doses. Furthermore, a reduction in the lenalidomide dose was required in a significant number of patients, highlighting the low tolerability of these older patients to therapy. This was especially true for those receiving lenalidomide–bortezomib-based triplets compared to doublets; however, the small sample size limits the ability to draw definitive conclusions. A third of the patients (n = 33) discontinued first-line therapy due to adverse events (AEs), with the presence of grade ≥ 3 AEs in 42% of patients (n = 14), likely underreported due to the retrospective nature of our study and the frequent lack of detailed documentation of all AEs. These high rates of serious AEs underscore the harsh impact of conventional MM therapies on the elderly [10].

A substantial number of patients experienced a worsening in their performance status, often associated with a lack of response to therapy, but also in patients who attained a significant response.

Infections, being the most frequently observed serious AEs, have not only affected quality of life but also increased the mortality risk. Notably, the treatment-related death rate was 9%, occurring despite the use of reduced doses of steroids and lenalidomide as well as the administration of granulocyte- colony stimulating factor (G-CSF), and infection prophylaxis with acyclovir and sulfamethoxazole-trimethoprim. This mortality rate is significantly greater than that reported in clinical prospective studies that evaluated various treatment regimens, including Velcade-Cyclophosphamide-Dexamethasone (VCD) [11,12,13]. This mainly reflects the differences between patients included in prospective clinical studies and those in our cohort. It is important to note that treatment regimens were also influenced by the Israeli health basket, which defined the available first-line agents during the study period. Newer agents, such as daratumumab, which was only added to the basket in 2023, are likely to be safer, potentially resulting in improved long-term outcomes. To support our statement, it should be noted that all octogenarian patients diagnosed and requiring therapy within the study period initiated an anti-MM therapy, even if presented with significant comorbidities and low performance status, a rate higher than expected for this age group.

In line with our results, a recent study including 110 octogenarian patients who were newly diagnosed with multiple myeloma assessed the outcomes among those patients. This study reported that 30% of patients received bortezomib-based treatment, with grade ≥ 3 adverse events occurring in 33% of patients during their first-line treatment, and 14% had grade 5 AEs over the course of their disease [14], highlighting the lower tolerance of these patients to treatment compared with their younger counterparts. This increased frailty underscores the need for comprehensive geriatric assessments before treatment and a personalized approach to minimize disabling AEs and prevent treatment discontinuation, which can worsen prognosis [15].

Despite these challenges, the objective response rate (ORR)of the entire cohort was 70%, which is encouraging and suggests that effective myeloma control can be achieved in octogenarians with careful management and appropriate treatment adjustments. A similar ORR was reported in clinical prospective studies exploring bortezomib in combination with steroids and alkylating agents in non-transplant-eligible patients, younger than evaluated in our cohort [16]. Nevertheless, the complete response (CR) rates in patients treated through clinical prospective studies were generally higher than that reported in our series [17], potentially due to the higher doses being administered and the better adherence to study treatment, which were feasible in patients eligible to be included in these studies [18,19,20,21]. Despite the low tolerance to therapy, which contributed to the short time to next treatment (TTNT), particularly in patients treated with triplets who had higher rates of treatment discontinuation and dose reductions, almost 70% of the patients in our cohort succeeded in receiving second-line therapy at disease progression, significantly greater than usually reported in real-world studies [22]. An EU international study evaluating the course of the disease in 4997 MM patients, age ≥ 18 years, treated across various centers in Europe showed that only 61% of patients succeeded in receiving second-line therapy, a lower proportion than reported in our octogenarian cohort, highlighting the remarkable success in providing salvage therapies in these octogenarian patients who are less likely to progress to subsequent therapies due to their age and comorbidities [23].

Despite their advanced age, with a median of 84 years, the overall survival (OS) of our cohort was relatively encouraging, reaching almost four years. This encouraging OS is likely to reflect our success in delivering subsequent therapies at any disease progression, even in these very elderly patients, giving them the chance to respond and adopting a “tailored” approach to improve their tolerance to therapy. This strategy demonstrates the significance of using an individualized approach. As expected, overall survival in patients who were older than 84 years and those presenting with high international staging system (ISS) and high Revised-ISS scores was short. This underscores the importance of individualized treatment approaches based on patient-specific risk factors, in addition to considering the patients’ comorbidities and general condition. Regardless of the wide range of novel treatment options, the ISS remains a strong predictor for OS in octogenarian patients and may help in guiding treatment strategies in this setting. The impact of high-risk factors on treatment decisions is likely to become even more significant, with the introduction of new agents that might offer superior results with a safer toxicity profile, promoting the administration of risk-adapted therapy. Interestingly, lung disease was associated with shorter OS, potentially due to an increased risk for lung infections, which often occur in these patients and were found to be responsible for some of the cases of treatment-related mortality (TRM). Interestingly, there were no significant differences in overall survival between patients treated with doublet versus triplet regimens, suggesting that at least with the anti-MM agents that were available to our patients during the study period, the addition of a third drug might not always translate to a survival benefit in this population. It seems that the similar OS between patients receiving triplets and doublets (despite the longer TTNT in those treated with doublets) is primarily attributed to patient selection to these regimens, whereas patients with favorable prognostic factors received doublets, and their higher-risk counterparts were treated with triplets. Nevertheless, the impact of using triplets vs. doublets cannot be fully defined due to the low number of patients and heterogeneity in treatment regimens and doses, reflecting the “tailored approach” employed in our octogenarian patients. Octogenarian patients may also benefit from local radiotherapy, especially for achieving effective pain control [24]. However, our series included only one patient that received irradiation: insufficient for making any conclusions regarding the role of radiotherapy in this population of patients.

## 5. Limitations

Our study faces several limitations. Firstly, the small sample size may limit the generalizability of the results. Octogenarian populations often present varied comorbidities and a higher degree of heterogeneity in health status, which can complicate the applicability of the findings to the broader elderly population. Secondly, the retrospective design of the study introduces potential biases, including selection bias and information bias. The data collected may not encompass all relevant patient information and the retrospective collection of clinical outcomes may not capture the full spectrum of adverse events or therapeutic responses. Thirdly and lastly, the rapid evolution of treatment modalities and supportive care techniques in multiple myeloma may outpace the relevance of these findings, emphasizing the need for ongoing research in this field, especially when considering the low number of patients that received a daratumumab-containing first-line therapy, the current standard-of-care treatment in MM patients [16,25,26,27,28,29].

## 6. Conclusions

Despite these limitations, this study significantly contributes to the existing literature by highlighting the real-world challenges and outcomes of treating octogenarian MM patients, emphasizing the clear need for personalized strategies that consider both the efficacy and tolerability of treatments, requiring dose adjustments and careful monitoring for adverse effects. Implementing comprehensive geriatric assessments, which are not routinely applied in daily practice, could help better identify patients who are at higher risk of treatment-related complications. There is also a need for more robust guidelines that specifically address the management of MM in the elderly, focusing on balancing efficacy with quality of life. Further research should aim to explore the long-term outcomes of various treatment regimens and the role of newer therapies and supportive care interventions that might be better tolerated in this age group.

## Figures and Tables

**Table 1:** Patient characteristics.

Gender		N (%)
	Male	54 (53.5)
	Female	47 (46.5)
**Age at diagnosis**		**Median (range)**
	All	84 (80–98.4)
	Male	84 (80–98.4)
	Female	84 (80–93.2)
**Prior Comorbidities**		**N (%)**
	NIDDM	33 (32.7)
	IHD	33 (32.7)
	CHF	15 (14.9)
	HTN	86 (85.1)
	CRF *	27 (26.7)
	TIA/CVA	5 (4.95)
	PVD	2 (1.98)
	Lung disease	17 (16.8)
	CTD	4 (3.96)
**Charlson comorbidity index**		**N (%)**
	4	33 (32.7)
	5	22 (21.8)
	6	21 (20.8)
	7	15 (14.9)
	8	4 (3.96)
	9	5 (4.95)
	11	1 (0.99)
**Follow-up period (months)**		**Median (range)**
		36 (0–141)
**ISS**		**N (%)**
	1	8 (7.9)
	2	23 (22.7)
	3	38 (37.6)
	Missing	32 (31.6)
**R-ISS**		**N (%)**
	1	6 (5.94)
	2	37 (36.6)
	3	20 (19.8)
	Missing	38 (37.6)
		**N (%)**
**Anemia**		50 (49.5)
**Lytic Lesions**		57 (56.4)
Acute Renal Failure *		19 (18.8)
**Cytogenetics High Risk**		30 (30.3)
**Prior MGUS**		15 (14.9)
**First-line treatment regimens**		**N (%)**
	Doublets	45 (44.5)
	Triplets	49 (48.5)
	Quadruplets	2 (1.98)
	Chemotherapy ± Steroids	5 (4.9)
	Radiotherapy **	1 (0.9)
	PI-containing regimen	88 (87.1)
	IMID-containing regimen	28 (27.7)
	PI-IMID-containing regimen	20 (19.8)

Abbreviations: NIDDM—non-insulin-dependent diabetes mellitus; IHD—ischemic heart disease; CHF—congestive heart failure; CRF—chronic renal failure; TIA—transient ischemic attack; PVD—peripheral vascular disease; CTD—connective tissue disease; ISS—International Staging System; R-ISS—Revised International Staging System; MGUS—monoclonal gammopathy of undetermined significance. * CCT was <30 for 50% (n = 51) including prior CRF and acute RF. ** Radiotherapy was given for pain control in combination with novel agents.

**Table 2:** Univariate analysis for factors predicting overall survival.

Variable	HR (Non-Adjusted)	95% CI	*p*-Value
Gender	1.57	0.97–2.53	0.064
HT	0.62	0.36–1.13	0.12
NIDDM	0.73	0.43–1.24	0.25
IHD	1.27	0.77–2.09	0.36
CHF	1.34	0.72–2.5	0.36
Lung Disease	1.8	1.02–3.15	0.044
ISS (3 vs. 1 + 2)	1.65	1.21–2.25	0.0016
R-ISS (3 vs. 1 + 2)	2.45	1.3–4.64	0.006
CRF	1.2	0.71–2.05	0.5
Prior malignancy	1.34	0.79–2.26	0.3
Prior VTE	0.83	0.261–2.66	0.76
AF	1.18	0.6–2.32	0.62
Lytic lesions	0.88	053–1.46	0.62
Cytogenetic Risk	1.51	0.79–2.89	0.21
Prior MGUS	1.33	0.7–2.56	0.4
Age group > 84 vs. 80–84 (yr)	1.72	1.07–2.78	0.03
Cytogenetic risk	1.51	0.79–2.89	0.213
Charlson index; ≥5 vs. lower	1.49	0.93–2.39	0.0497

Abbreviations: HT—hypertension; NIDDM—non-insulin-dependent diabetes mellitus; IHD—ischemic heart disease; CHF—congestive heart failure; ISS—International Staging System; R-ISS—Revised International Staging System; RF—renal failure; VTE—venous thromboembolism; AF—atrial fibrillation; MGUS—monoclonal gammopathy of undetermined significance.

## Data Availability

The data collected, used, and analyzed in this study can be made available by the corresponding author upon request.

## Supplementary Materials

The following supporting information can be downloaded at: https://www.mdpi.com/article/10.3390/cancers16193329/s1, Table S1: First line treatment regimens, Table S2: Second line treatment regimens, Table S3: Univariate analysis for factors predicting time to second line therapy, Table S4: Multivariate analysis for factors predicting Overall Survival.
