# Supplementary material for: The Outcome of Octogenarian Patients with Multiple Myeloma Treated Outside Clinical Studies, Focusing on Tolerability and Efficacy of Treatment
Source: Cancers (Basel). 2024 Sep 29;16(19):3329. doi: 10.3390/cancers16193329 (PMC11475655; doi:10.3390/cancers16193329)
Supplement: Supplementary file 1 [file cancers-16-03329-s001.zip › cancers-3192362-supplementary.pdf]

Supplementary Materials: The Outcome of Octogenarian Patients with Multiple Myeloma Treated Outside Clinical Studies, Focusing on Tolerability and Efficacy of Treatment

Dana Amsterdam [1,2,*], Ori Grossberger [1,2], Natan Melamed [1], Dor Shpizer [1,2], Svetlana Trestman [1,2], Tamir Shragai [1,2], Yael C. Cohen [1,2] and Irit Avivi [1,2,*]

[1] Hematology Division, Tel Aviv Sourasky Medical Center, Tel Aviv Post code 6423906, Israel; orig@tlvmc.gov.il (O.G.); natanm@tlvmc.gov.il (N.M.); shpitzerd@gmail.com (D.S.); svetlanat@tlvmc.gov.il (S.T.); tamirsh@tlvmc.gov.il (T.S.); yaelcoh@tlvmc.gov.il (Y.C.C.)
[2] Faculty of Medicine, Tel Aviv University, Post code 6139001, Israel
* Correspondence: danaams@tlvmc.gov.il (D.A.); iritavi@tlvmc.gov.il (I.A.)

Table S1. First line treatment regimens.

First line treatment regimens	Number (%)
**Doublets**	45 (44.5)
VD	38 (37.6)
RD	6 (5.9)
THAL-D	1 (0.9)
**Triplets/ Quadruplets**	51 (50.4)
VCD	22 (21.8)
VRD	18 (17.8)
VTD	2 (1.9)
Dara-VD	5 (4.9)
Dara-VRD	2 (1.9)
KRD	1 (0.9)
IXA-VD	1 (0.9)
Containing Bortezomib	88 (87.1)
Containing Lenalidomide	27 (26.7)
Chemotherapy	5 (4.9)

Abbreviations: VD- velcade. RD-revlimid. VCD- velcade, Cyclophosphamide, Dexamethasone. VRD- Velcade, Revlimid, Dexamethasone. Rd- Revlimid. DARA VD- Daratumumab, Velcade. VTD- Velcade, Thalidomide, Dexamethasone. DARA VRD- Daratumumab, Velcade, Revlimid, Dexamethasone. KRD- Kyprolis, Revlimid, IXA-V- Ixazomib, Velcade. THAL- Thalidomide.

Table S2. Second line treatment regimens.

Second line treatment regimens	Number (%)
**Doublets**	39 (57)
VD	12 (17.6)
RD	18 (26.4)
DARA	9 (13.2)
**Triplets/ Quadruplets**	29 (42)
VCD	2 (2.9)
VRD	5 (7.3)
VTD	2 (2.9)
Dara-VD	2 (2.9)
Dara-VCD	1 (1.4)
CARF-R-DARA	1 (1.4)
IXA-RD	5 (7.3)
RD-DARA	9 (13.2)

RD-ELO	1 (1.4)
Pom-DARA	1 (1.4)
**ANTI MM REGIMEN**	68 (100)
PI based (including VRD)	24 (35.2)
IMID based	34 (50)

Abbreviations: VD- velcade. RD-revlimid. DARA- daratumumab. VCD- velcade, Cyclophosphamide, Dexamethasone. VRD- Velcade, Revlimid, Dexamethasone. Rd- Revlimid. DARA VD- Daratumumab, Velcade. VTD- Velcade, Thalidomide, Dexamethasone. DARA VCD- Daratumumab, Cyclophosphamide, Velcade, Dexamethasone. CARF-R-DARA- Carfilzomib, Revlimid, daratumumab. IXA- RD- Ixazomib, Revlimid. RD-DARA- Revlimid-Daratumumab. RD-ELO- Revlimid, elotuzumab. POM-DARA- Pomalidomide- Daratumumab.

Table S3. Univariate analysis for factors predicting time to second line therapy.

Variable	HR (non-adjusted)	95% CI	P-Value
Gender	1.3252	0.8159-2.152	0.25
HT	1.412	0.675-2.956	0.36
NIDDM	0.8289	0.48-1.41	0.489
IHD	1.063	0.63-1.79	0.818
CHF	0.98	0.49-1.99	0.975
Lung Disease	0.99	0.52-1.89	0.978
ISS (1+2 vs 3)	1.4	0.79 -2.5	0.256
R-ISS (1+2 vs 2)	1.2575	0.6537-2.419	0.49
Renal failure	0.82	0.4587-1.451	0.49
Prior malignancy	0.9505	0.5534-1.633	0.854
Prior VTE	1.04142	0.3264-3.323	0.945
Lytic lesions	1.367	0.8189-2.281	0.232
Cytogenetic Risk	1.7265	0.919-3.244	0.09
Prior MGUS	1.08825	0.5552-2.133	0.805
Age group (years) 80-84 VS 84.1 -89	0.7096	0.4383-1.149	0.163
Doublets vs Triplets	2.2	1.302-3.701	0.003
Albumin <=3.5 vs Albumin > 3.5 (gr/dl)	1.4025	0.7078-2.779	0.33
Creatinine <=1.3 vs Creatinine > 1.3 (mg/dl)	0.7929	0.4639-1.355	0.4

Abbreviations: HT-hypertension; NIDDM- non-insulin dependent diabetes mellitus; IHD- ischemic heart disease; CHF -congestive heart failure; ISS-International Staging System; R-ISS-revised International Staging System; VTE- Venous Thromboembolism; MGUS- Monoclonal gammopathy of undetermined significance.

Table S4. Multivariate analysis for factors predicting Overall Survival.

Variable	HR	95%CI	p
Age	1.57305	0.7765-3.187	0.2085
Respiratory disease	1.49875	0.6759-3.323	0.3193
ISS	2.24416	0.9307-5.411	0.0718
R-ISS	1.05775	0.6792-1.647	0.8038

Abbreviations: ISS-International Staging System; R-ISS-revised International Staging System.
